# Draft Genome Sequences of Cronobacter muytjensii Cr150, Cronobacter turicensis Cr170, and Cronobacter sakazakii Cr611

**DOI:** 10.1128/MRA.00660-20

**Published:** 2020-10-29

**Authors:** Gal Zizelski Valenci, Mor Rubinstein, Reuven Afriat, Shira Rosencwaig, Zeev Dveyrin, Efrat Rorman, Israel Nissan

**Affiliations:** a Ministry of Health, National Public Health Laboratory, Tel Aviv, Israel; University of Maryland School of Medicine

## Abstract

The sequencing and bioinformatics analyses of isolates Cr150, Cr170, and Cr611 from powdered infant formula indicate that the three strains represent new members in the Cronobacter muytjensii, Cronobacter turicensis, and Cronobacter sakazakii groups, respectively.

## ANNOUNCEMENT

*Cronobacter* spp. are a group of opportunistic bacteria associated with severe and potentially life-threatening diseases (1–3). In order to expand our knowledge about this group of pathogens, multiple confirmed *Cronobacter* samples were sequenced and analyzed. One of them is Cronobacter sakazakii Cr268 (4). Here, we present the genomes of Cronobacter muytjensii Cr150, Cronobacter turicensis Cr170, and Cronobacter sakazakii Cr611.

The three strains were isolated and identified exactly as indicated in ISO 22964:2006 (5). Matrix-assisted laser desorption ionization–time of flight mass spectrometry (MALDI-TOF MS) analysis (6) indicates that Cr150, Cr170, and Cr611 belong to the Cronobacter sakazakii group. The isolates were kept frozen (−80°C) until their revival. Two typical colonies were collected from each isolate using an inoculating loop and suspended in 400 μl of saline followed by two washes and resuspension in 200 μl of saline. DNA was isolated from overnight culture using a MagNA pure compact robot (Roche) following the manufacturer’s instructions. Paired-end libraries were generated using an Illumina Nextera DNA Flex DNA library preparation kit according to Illumina protocols. For sequencing, we utilized the Illumina MiSeq platform using a 250-bp paired-end read kit v2.

All bioinformatic analyses were performed using the PATRIC v3.5.36 platform (7) with default parameters, unless otherwise noted. The quality of the raw reads was checked using the FASTQ utilities of PATRIC. The total numbers of reads for Cr150, Cr170, and Cr611 were 2,604,470, 2,086,159, and 2,053,349, respectively. *De novo* assembly of the reads was performed using SPAdes (8) as part of the PATRIC Comprehensive Genome Analysis Service. The assembly and annotation information is detailed in Table 1. Genome annotation was performed with the Rapid Annotations using Subsystems Technology tool kit (RASTtk) (9) and the NCBI Prokaryotic Genome Annotation Pipeline (PGAP) at the National Center for Biotechnology Information (NCBI). CRISPR/Cas systems were identified using CRISPRCasFinder (10) (Table 1). In order to identify the closest homologue in the database, as well as the as the closest high-quality representative genome at the NCBI, we utilized the Similar Genome Finder Service, which uses the Mash/MinHash algorithm (11), and the average nucleotide identity (ANI) calculator (12) to estimate average nucleotide identity (Table 1). We also performed taxonomic classification using Kraken v2 (13), confirming strain taxonomy. The multilocus sequence type (MLST) profile was determined using the MLST 2.0 server at the Center for Genomic Epidemiology (genomicepidemiology.org) (14) (Table 1).

**TABLE 1:** Assembly, annotation, and MLST details of the C. sakazakii Cr611, *C. muytjensii* Cr150, and *C. turicensis* Cr170 genomes

Genomic feature	Data for strain:		
	Cr170	Cr150	Cr611
Assembly details^*a*^			
No. of contigs	27	37	18
*N*_50_ contig length (bp)	534,952	592,056	525,942
GC content (%)	57.42	57.23	56.98
Genome length (bp)	4,498,812	4,613,086	4,443,709
Properly mapped reads (%)	99.84	99.84	99.82
Median base coverage (×)	121	105	105
Contig *L*_50_	3	4	4
Genome annotation			
No. of protein coding sequences	4,281	4,472	4,245
No. of tRNAs	75	77	76
No. of rRNAs	10	10	10
CRISPR			
No. of CRISPR spacers	27	80	19
No. of CRISPR repeats	29	83	21
CRISPR/Cas system	CAS-TypeIE	CAS-TypeIF	CAS-TypeIE
CRISPR/Cas genes	Cas1, Cas2, Cas5, Cas6, Cas7, Cse1, Cse2	Cas1, Cas3-Cas2, Csy1, Csy2, Csy3, Csy6	Cas1, Cas2, Cas5, Cas6, Cas7, Cse1, Cse2
Similar Genome Finder Service (Mash/MinHash)			
Most similar genome in the database	*Cronobacter turicensis* cro3064B1 (GenBank accession no. NRJM01000000)	*Cronobacter muytjensii* MOD1-Md1s (GenBank accession no. MSAE00000000)	*Cronobacter sakazakii* CS-30 (GenBank accession no. QISO01000000)
Most similar genome K-mer count	651/1,000	651/1,000	574/1,000
Average nucleotide identity for most similar genome in the database (%)	98.74	98.96	98.23
Closest high-quality representative genome at NCBI	*Cronobacter turicensis* z3032 (GenBank accession no. FN543093)	*Cronobacter dublinensis* 1210 (GenBank accession no. CAKZ00000000)	*Cronobacter sakazaki*i ATCC 29544 (BioProject no. PRJNA276173)
Closest high-quality representative genome K-mer count	517/1,000	134/1,000	529/1,000
MLST			
Locus *atpD*			
Identity (%)	100	100	100
Coverage (%)	100	100	100
Alignment length (bp)	390	390	390
Allele length (bp)	390	390	390
Allele	*atpD_68*	*atp_35*	*atpD_3*
Locus *fusA*			
Identity (%)	100	100	100
Coverage (%)	100	100	100
Alignment length (bp)	438	438	438
Allele length (bp)	438	438	438
Allele	*fusA_5*	*fusA_35*	*fusA_14*
Locus *glnS*			
Identity (%)	100	99.449	99.7245
Coverage (%)	100	100	100
Alignment length (bp)	363	363	363
Allele length (bp)	363	363	363
Allele	*glnS_42*	*glnS_176*^*b*^	*glnS_9*^*b*^
Locus *gltB*				
Identity (%)	99.6055	99.211	100
Coverage (%)	100	100	100
Alignment length (bp)	507	507	507
Allele length (bp)	507	507	507
Allele	*gltB_62*^*b*^	*gltB_242*^*b*^	*gltB_109*
Locus *gyrB*			
Identity (%)	100	100	99.32537
Coverage (%)	100	100	100
Alignment length (bp)	402	402	402
Allele length (bp)	402	402	402
Allele	*gyrB_60*	*gyrB_45*	*gyrB_43*^*b*^
Locus *infB*			
Identity (%)	100	100	100
Coverage (%)	100	100	100
Alignment length (bp)	441	441	441
Allele length (bp)	441	441	441
Allele	*infB_150*	*infB_4*	*infB_56*
Locus *pps*			
Identity (%)	99.596	99.596	100
Coverage (%)	100	100	100
Alignment length (bp)	495	495	495
Allele length (bp)	495	495	495
Allele	*pps_156*^*b*^	*pps_75*^*b*^	*pps_13*

a The *N*_50_ length is defined as the shortest sequence length at 50% of the genome, and the *L*_50_ count is defined as the smallest number of contigs whose length sum produces *N*_50_.

b Novel allele.

Importantly, the MLSTs for these three strains are unknown, with two novel alleles for isolates Cr611 and Cr170 and three novel alleles for isolate Cr150. The closest homologue for each strain identified in the database possesses a low k-mer similarity (15) (Table 1). Notably, although Cr150 was classified using Kraken v2 as *C. muytjensii*, the Mash/MinHash algorithm identified Cronobacter dublinensis 1210 (GenBank accession number CAKZ00000000) as the closest high-quality representative genome in NCBI, indicating that the NCBI database does not contain a high-quality representative genome for *C. muytjensii*.

In conclusion, strains Cr150, Cr170, and Cr611 represent new members in the *C. muytjensii*, *C. turicensis*, and C. sakazakii groups, respectively.

### Data availability.

The sequences are now available to the public at the NCBI (BioProject number PRJNA636126).
